# Diagnostic performance of the expanded National Institutes of Health Stroke Scale for the assessment of anterior and posterior circulation strokes

**DOI:** 10.3389/fneur.2026.1788839

**Published:** 2026-09-14

**Authors:** Stefano Forlivesi, Andrea Balbarini, Guido Bigliardi, Roberto D’Amico, Francesca Rosafio, Laura Vandelli, Maria Luisa Dell’Acqua, Federica Casoni, Stefano Meletti, Milena Cavazzuti, Andrea Zini

**Affiliations:** 1 IRCCS Istituto delle Scienze Neurologiche di Bologna, Department of Neurology and Stroke Center, Maggiore Hospital, Bologna, Italy; 2 Local Health Authority of Modena, General Practice, Modena, Italy; 3 Stroke Unit, Department of Neuroscience, Ospedale Civile di Baggiovara, Modena University Hospital, Modena, Italy; 4 Unit of Statistical and Methodological Support to Clinical Research, Azienda Ospedaliero-Universitaria, Modena, Italy; 5 Department of Medical and Surgical Sciences, University of Modena and Reggio Emilia, Modena, Italy; 6 Local Health Authority of Modena, Healthcare Management, Modena, Italy; 7 Department of Biomedical, Metabolic, and Neural Sciences, University of Modena and Reggio-Emilia, Modena, Italy

**Keywords:** acute stroke, diagnostic performance, NIHSS (National Institue of Health Stroke Scale), posterior circulation stroke, prognosis

## Abstract

**Background:**

The main limitation of the National Institutes of Health Stroke Scale (NIHSS) is the underestimation of clinical severity in posterior circulation (PC) strokes, which has been hypothesized to contribute to diagnostic delay and to reduce eligibility for reperfusion treatments in some patients. We aimed to evaluate the diagnostic performance of a previously developed modified version of the NIHSS, the Expanded NIHSS (e-NIHSS), for clinical assessment and outcome prediction in patients with anterior circulation (AC) and PC strokes.

**Methods:**

Prospective observational study of 249 consecutive stroke patients admitted to a Comprehensive Stroke Center during 1 year. NIHSS and e-NIHSS were administered by two independent examiners within 48 h of onset. The primary outcome measure was an unfavorable outcome, defined as a modified Rankin Scale score of 3 to 6 at 3 months. The discriminative performance of the e-NIHSS to predict unfavorable outcome was evaluated by calculating the area under the receiver operating characteristic curve (AUC-ROC).

**Results:**

The inter-rater reliability of the e-NIHSS between the two independent observers was excellent (intraclass correlation coefficient, 0.992 [95% CI 0.990–0.994]). The mean e-NIHSS score was higher than the mean classical NIHSS score (*p* < 0.001). An unfavourable outcome at 3 months was detected in 113 (45%) patients. The e-NIHSS showed a discriminative performance to predict unfavorable functional outcome comparable to the classical NIHSS for the total amount of strokes (AUC-ROC of the e-NIHSS for the first observer, 0.867 [95% CI, 0.823–0.911]; AUC-ROC of the e-NIHSS for the second observer, 0.871 [95% CI, 0.827–0.914]; AUC-ROC of the classical NIHSS, 0.849 [95% CI, 0.802–0.897]), AC strokes (AUC-ROC of the e-NIHSS for the first observer, 0.886 [95% CI, 0.841–0.932]; AUC-ROC of the e-NIHSS for the second observer, 0.892 [95% CI, 0.848–0.936]; AUC-ROC of the classical NIHSS, 0.870 [95% CI, 0.821–0.920]), and PC strokes (AUC-ROC of the e-NIHSS for the first observer, 0.800 [95% CI, 0.685–0.916]; AUC-ROC of the e-NIHSS for the second observer, 0.791 [95% CI, 0.672–0.910]; AUC-ROC of the classical NIHSS, 0.804 [95% CI, 0.689–0.920]). Net reclassification improvement, integrated discrimination improvement, and decision curve analysis showed a modest apparent incremental prognostic benefit of the e-NIHSS over the classical NIHSS in the whole cohort and in AC strokes, but not in PC strokes.

**Conclusion:**

Compared to the classical NIHSS, the e-NIHSS showed a similar discriminative performance to predict 3-month functional outcome, capturing and quantifying additional PC-relevant clinical signs not fully represented in the classical NIHSS, with excellent inter-rater reliability.

## 1 Introduction

The National Institutes of Health Stroke Scale (NIHSS) is the most widespread clinical scale used for the assessment of patients presenting with acute stroke (1). It evaluates 11 neurologic functions divided into 15 items, with a total score ranging from 0 (normal neurological examination) to 42 (unresponsive coma). It provides a standardized evaluation of stroke signs and symptoms, and its merits include simplicity, quickness, good agreement between clinicians, and strong prognostic value thanks to correlation with 3-month functional outcome (2–4).

The main limitation of the NIHSS is the underestimation of clinical severity in posterior circulation (PC) strokes, and previous studies showed a lower NIHSS threshold for unfavorable outcome in patients with PC stroke compared with patients with anterior circulation (AC) stroke (5–7). As a matter of fact, the final score strongly depends on signs and symptoms of AC stroke, whereas many clinical manifestations related to the vertebrobasilar system (i.e., diplopia, dysphagia, gait instability, hearing loss, and nystagmus) receive no score. This may cause diagnostic delay of PC strokes and limit access to reperfusion treatments. In fact, recent evidence showed no benefit of intravenous thrombolysis (IVT) with either alteplase or tenecteplase in patients with mild (i.e., NIHSS 0–5) nondisabling stroke (8–10). However, clinical assessment with the classical NIHSS may not completely capture disabling symptoms of PC stroke, and exclude from treatment patients who might potentially benefit from IVT. Moreover, in the last few years, large clinical trials demonstrated a significant benefit of endovascular treatment (EVT) in patients with moderate-to-severe PC stroke (11), and a recent multicenter cohort study suggested that even mild PC strokes may benefit from EVT (12). For these reasons, in patients with acute stroke, a rapid detection of PC signs and symptoms would be highly desirable.

We previously developed an expanded version of the NIHSS, the Expanded NIHSS (e-NIHSS), applicable to both AC and PC strokes, with increased sensitivity for PC strokes (13). The scale consists of the same 15 items of the NIHSS, with 5 categories added to the existing items 4, 6, and 11 to explore signs and symptoms of PC stroke. In the e-NIHSS item 4, nystagmus and Horner syndrome are both scored 1. In the e-NIHSS item 6, lingual palsy and asymmetry in velum elevation are both scored 3. In the e-NIHSS item 11, latero- or retropulsion in the sitting position is scored 2, while instability in the Romberg position is scored 1 (13).

The use of the e-NIHSS in clinical practice could improve the triage of patients with suspected stroke, thanks to the rapid recognition of PC signs and symptoms, ensuring immediate care by the stroke team and, potentially, more timely engagement with acute-phase treatment pathways.

The main limitations of the study were the small cohort dimension and the lack of assessment of the correlation between the e-NIHSS score and the 3-month functional outcome.

In the present study, we aimed to evaluate the diagnostic performance of the e-NIHSS for clinical assessment and outcome prediction in a larger cohort of patients with AC and PC strokes.

## 2 Materials and methods

We conducted a prospective observational study of consecutive acute stroke patients admitted to a Comprehensive Stroke Center during 1 year, within 48 h of symptom onset.

Stroke was clinically diagnosed by expert neurologists and confirmed by neuroimaging. Computer tomography and/or magnetic resonance imaging were used.

The collected data included age, gender, time of symptoms onset, type of stroke (i.e., ischemic or hemorrhagic stroke), reperfusion treatments (i.e., IVT and/or EVT), stroke territory (i.e., AC or PC stroke defined by neuroimaging), stroke subtypes according to the Bamford classification (14), stroke etiology according to the Trial of ORG 10172 in Acute Stroke Treatment (TOAST) criteria (15), and comorbidities.

In our center, the indication for reperfusion treatments is given according to the most recent national and international guidelines. As regards areas of uncertainty, including a low NIHSS score, the final decision is taken by the treating neurologist and interventional neuroradiologist based on the individual characteristics of the patient.

NIHSS and e-NIHSS were administered to every patient by two independent examiners with NIHSS certification within 48 h of symptom onset, and registered in the patient’s medical record. Time of symptom onset was recorded for every patient, primarily to determine eligibility for reperfusion therapies and for study inclusion within the 48-h window; however, the exact time of NIHSS/e-NIHSS assessment relative to symptom onset was not systematically captured as a separate discrete variable, precluding calculation of the exact onset-to-assessment interval for each patient.

Three-month functional outcome was assessed by clinical examination or telephone interview using the modified Rankin Scale (mRS). The primary outcome measure was an unfavorable outcome, defined as an mRS score of 3 to 6 at 3 months.

### 2.1 Statistical analysis

We performed all statistical analyses using IBM SPSS Statistics version 24.0 and R version 4.5.3. Continuous variables were reported as mean (standard deviation) or median (interquartile range) values. Proportions were calculated for categorical variables, dividing the number of events by the total number excluding missing/unknown cases. We examined differences in continuous variables using the Student’s t-test, the Kruskal-Wallis one-way analysis of variance, or the Mann–Whitney U-test when appropriate. Differences between proportions were assessed by use of Fisher’s exact test or χ^2^ test when appropriate.

We used specific validation criteria for scales. The inter-rater reliability of the e-NIHSS total score between the two independent observers was assessed using the intraclass correlation coefficient (ICC), based on a two-way random-effects, absolute-agreement, single-measures model [ICC(2,1)], with ICC < 0.50 indicating poor reliability, 0.50–0.75 moderate, 0.75–0.90 good, and >0.90 excellent reliability. The validity of the e-NIHSS was assessed by comparing it with the NIHSS through Spearman’s Rank correlation coefficient. The statistical weight on the final score of the new categories in the e-NIHSS was assessed using the Wilcoxon test.

We tested the independent association between variables and the outcome measure with a multivariate logistic regression model, including all variables that showed a significant association in the univariate analysis.

The discriminative performance of the e-NIHSS to predict unfavorable functional outcome at 3 months was evaluated by calculation of the area under the receiver operating characteristic curve (AUC-ROC), with two-sided 95% confidence intervals (CIs), ranging from 0.5 to 1, with values ≥0.8 considered as good predictive ability. We compared the AUC-ROCs of the classical NIHSS and the e-NIHSS using the DeLong test. We established statistical significance at two-tailed 0.05 level (*p* < 0.05).

To further characterize the added value of the e-NIHSS over the classical NIHSS in predicting 3-month unfavorable outcome, we performed: continuous net reclassification improvement (NRI), which quantifies the proportion of patients whose predicted risk is appropriately reclassified upward (for patients with the outcome) or downward (for patients without the outcome) by the e-NIHSS relative to the NIHSS; integrated discrimination improvement (IDI), calculated as the difference between the two models’ discrimination slopes (the difference between the mean predicted probability among patients with and without the outcome); the Hosmer–Lemeshow goodness-of-fit test, to assess model calibration; and decision curve analysis (DCA), which quantifies the net clinical benefit of using model-predicted probabilities to guide a hypothetical treatment decision across a range of threshold probabilities, referenced against the “treat all” and “treat none” strategies. These four analyses were performed separately for each observer’s e-NIHSS score, and separately for the whole cohort and for the AC and PC stroke subgroups; 95% confidence intervals for NRI and IDI were obtained from 1,500 bootstrap resamples. Both the NIHSS- and e-NIHSS-based logistic regression models were refit in each resampled dataset. As both models were developed and evaluated in the same single cohort, the reported discrimination, reclassification, calibration, and net-benefit estimates represent apparent, in-sample performance rather than internally or externally validated performance, and the results of these four analyses should accordingly be regarded as exploratory.

## 3 Results

Overall, 249 consecutive patients with acute stroke were included in the study (Figure 1). Thirty (12%) patients were affected by hemorrhagic stroke, whereas 219 (88%) by ischemic stroke. A total of 194 (78%) patients had an AC stroke, and 55 (22%) had a PC stroke defined by neuroimaging. The clinical characteristics of the patients are provided in Table 1.

**Figure 1:**
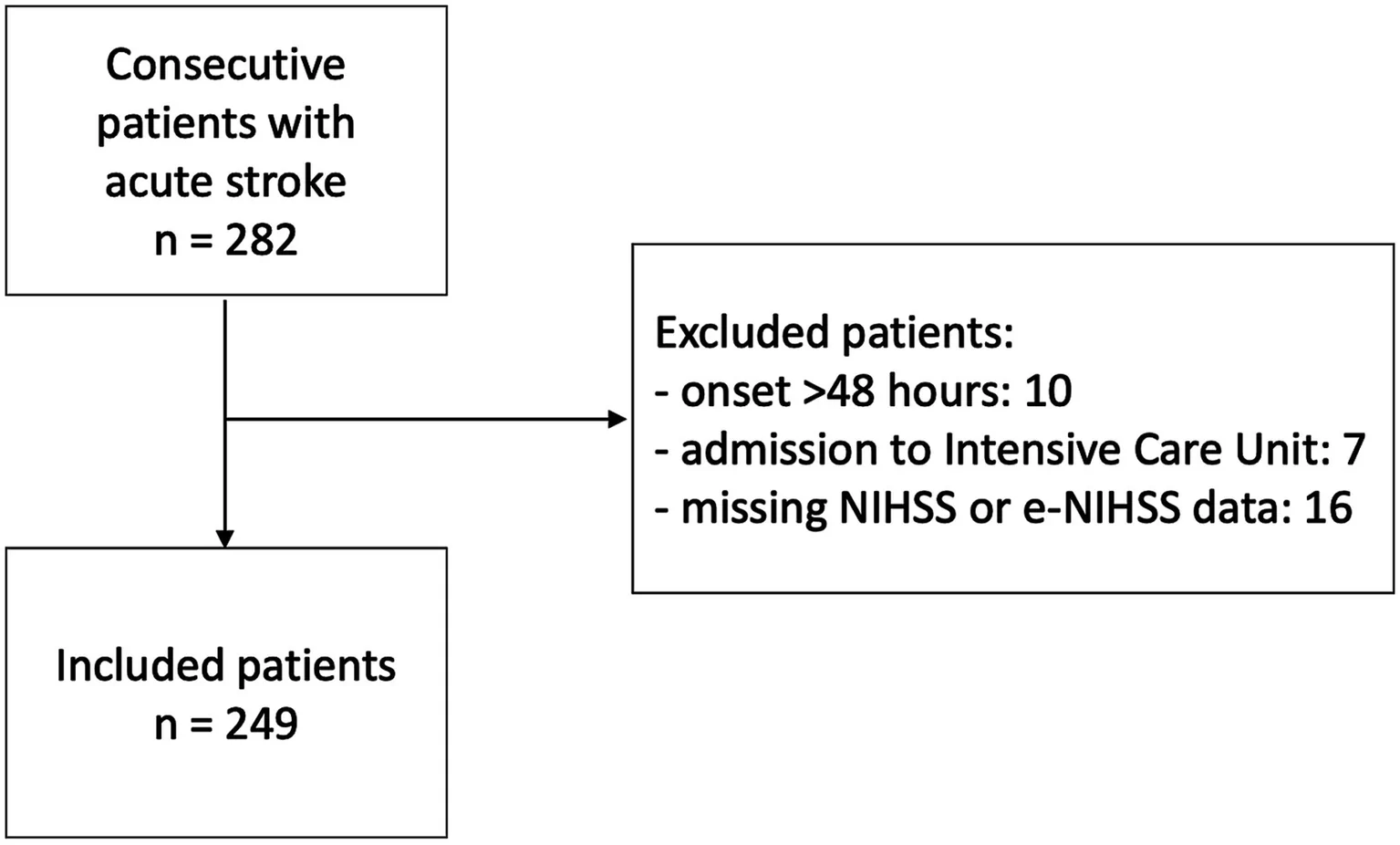
Flowchart of patients’ enrollment.

**Table 1:** Characteristics of patients with anterior circulation and posterior circulation stroke.

Characteristics	All patients (*n* = 249)	AC stroke (*n* = 194)	PC stroke (*n* = 55)	*p* value
Age (years), median (IQR)	74.0 (64.0–82.0)	74.5 (64.0–82.0)	74.0 (63.5–81.0)	0.806
Male sex, *n* (%)	134 (53.8)	105 (54.1)	29 (52.7)	0.855
Atrial fibrillation, *n* (%)	71 (28.5)	57 (29.4)	14 (25.5)	0.569
Hypertension, *n* (%)	200 (80.3)	156 (80.4)	44 (80.0)	0.946
Diabetes mellitus, *n* (%)	48 (19.3)	30 (15.5)	18 (32.7)	**0.004**
Hypercholesterolemia, *n* (%)	104 (41.8)	73 (37.6)	31 (56.4)	**0.013**
Current smoking, *n* (%)	59 (23.7)	49 (25.3)	10 (18.2)	0.276
Coronary artery disease, *n* (%)	44 (17.7)	33 (17.0)	11 (20.0)	0.608
Previous stroke/TIA, *n* (%)	17 (6.8)	13 (6.7)	4 (7.3)	0.882
NIHSS, mean (SD)	6.21 (6.895)	6.97 (7.320)	3.51 (4.163)	**0.001**
e-NIHSS observer 1, mean (SD)	7.58 (7.493)	8.27 (7.977)	5.15 (4.755)	**0.006**
e-NIHSS observer 2, mean (SD)	7.57 (7.384)	8.24 (7.881)	5.20 (4.588)	**0.007**
Hemorrhagic stroke, *n* (%)	30 (12.0)	26 (13.4)	4 (7.3)	0.218
IVT, n (%)	112 (45.0)	86 (44.3)	26 (47.3)	0.699
EVT, n (%)	78 (31.3)	68 (35.1)	10 (18.2)	**0.017**

AC, Anterior Circulation; e-NIHSS, Expanded National Institutes of Health Stroke Scale; EVT, Endovascular Treatment; IQR, Interquartile Range; IVT, Intravenous Thrombolysis; NIHSS, National Institutes of Health Stroke Scale; PC, Posterior Circulation; SD, Standard Deviation; TIA, Transient Ischemic Attack. Values in bold are statistically significant.

Inter-rater reliability of the e-NIHSS total score was excellent (ICC 0.992, 95% CI 0.990–0.994). Item-level reliability ranged from 0.826 to 0.970 across the 15 items (Table 2). Inter-rater reliability of the e-NIHSS total score was similarly excellent when stratified by circulation territory (ICC 0.993, 95% CI 0.990–0.995 for AC stroke; ICC 0.985, 95% CI 0.975–0.991 for PC stroke).

**Table 2:** Inter-rater reliability of the total score and of each item of the e-NIHSS between the two independent observers.

e-NIHSS	ICC (95% CI)
1. Level of consciousness	0.907 (0.882–0.927)
2. Questions	0.970 (0.961–0.976)
3. Commands	0.905 (0.881–0.926)
4. Best gaze	0.852 (0.814–0.883)
5. Visual field	0.940 (0.924–0.953)
6. Facial palsy	0.966 (0.957–0.974)
7. Motor arm (right side)	0.959 (0.947–0.968)
8. Motor arm (left side)	0.962 (0.952–0.970)
9. Motor leg (right side)	0.926 (0.907–0.942)
10. Motor leg (left side)	0.940 (0.924–0.953)
11. Limb ataxia	0.949 (0.935–0.960)
12. Sensory	0.850 (0.811–0.881)
13. Best language	0.961 (0.951–0.970)
14. Dysarthria	0.903 (0.877–0.923)
15. Extinction and inattention	0.826 (0.781–0.861)
Total score	0.992 (0.990–0.994)

CI, Confidence Interval; e-NIHSS, Expanded National Institutes of Health Stroke Scale; ICC, intraclass correlation coefficient; NIHSS, National Institutes of Health Stroke Scale.

The correlation between the two examiners obtained excellent agreement between e-NIHSS and NIHSS: e-NIHSS/NIHSS, 0.957 (observer 1); and e-NIHSS/NIHSS, 0.955 (observer 2).

The mean e-NIHSS score was higher than the mean classical NIHSS score: the mean e-NIHSS score was 7.58 (standard deviation, 7.493) for the first observer and 7.57 (standard deviation, 7.384) for the second observer, while the mean classical NIHSS score was 6.21 (standard deviation, 6.895). In AC strokes, the mean e-NIHSS score was 8.27 (standard deviation, 7.977) for the first observer and 8.24 (standard deviation, 7.881) for the second observer, while the mean classical NIHSS score was 6.97 (standard deviation, 7.320). In PC strokes, the mean e-NIHSS score was 5.15 (standard deviation, 4.755) for the first observer and 5.20 (standard deviation, 4.588) for the second observer, while the mean classical NIHSS score was 3.51 (standard deviation, 4.163). All the differences between the e-NIHSS for observer 1 and the classical NIHSS, and between the e-NIHSS for observer 2 and the classical NIHSS were statistically significant (*p* < 0.001), weighted by the expanded items.

The e-NIHSS score was higher than the classical NIHSS score in 187 (75%) patients, whereas the score was the same in 62 (25%) patients, for both observer 1 and observer 2. Twenty-four patients for observer 1 and 26 patients for observer 2 passed from an NIHSS <6 to an e-NIHSS ≥6.

Nystagmus and/or Horner’s syndrome (expanded item 4) were detected more frequently in PC stroke than in AC stroke. In contrast, Romberg imbalance and trunk ataxia (expanded item 11) were almost equally present in both AC and PC stroke (Table 3).

**Table 3:** e-NIHSS items 4, 6, and 11 in patients with anterior circulation and posterior circulation stroke.

e-NIHSS	Observer 1				Observer 2			
	All patients (*n* = 249)	AC stroke (*n* = 194)	PC stroke (*n* = 55)	*p* value	All patients (n = 249)	AC stroke (*n* = 194)	PC stroke (*n* = 55)	*p* value
Expanded item 4: nystagmus and/or Horner’s syndrome, *n* (%)	17 (6.8)	8 (4.1)	9 (16.4)	0.004	17 (6.8)	7 (3.6)	10 (18.2)	0.001
Expanded item 6: deficit of IX and/or XII cranial nerves, *n* (%)	34 (13.7)	23 (11.9)	11 (20.0)	0.121	39 (15.7)	28 (14.4)	11 (20.0)	0.316
Expanded item 11: Romberg imbalance, *n* (%)	22 (8.8)	17 (8.8)	5 (9.1)	1.000	23 (9.2)	17 (8.8)	6 (10.9)	0.604
Expanded item 11: trunk ataxia, *n* (%)	116 (46.6)	89 (45.9)	27 (49.1)	0.673	116 (46.6)	90 (46.4)	26 (47.3)	0.908

AC, Anterior Circulation; e-NIHSS, Expanded National Institutes of Health Stroke Scale; PC, Posterior Circulation.

An unfavourable outcome at 3 months was detected in 113 (45%) patients, of which 89 with AC stroke and 24 with PC stroke. Mortality rate at 90 days was 7.6% (19 patients) for all strokes, 7.7% (15 patients) for AC strokes, and 7.3% (4 patients) for PC strokes. In the multivariate logistic regression analyses, both NIHSS and e-NIHSS showed an independent association with unfavorable functional outcome at 3 months for all strokes, AC strokes, and PC strokes (Supplementary Tables 1–3).

The e-NIHSS showed a discriminative performance to predict unfavorable functional outcome comparable to the classical NIHSS for the total amount of strokes (AUC-ROC of the classical NIHSS, 0.849 [95% CI 0.802–0.897]; AUC-ROC of the e-NIHSS for the first observer, 0.867 [95% CI 0.823–0.911, *p* = 0.06]; AUC-ROC of the e-NIHSS for the second observer, 0.871 [95% CI 0.827–0.914, *p* = 0.02]) (Figure 2A), AC strokes (AUC-ROC of the classical NIHSS, 0.870 [95% CI 0.821–0.920]; AUC-ROC of the e-NIHSS for the first observer, 0.886 [95% CI 0.841–0.932, *p* = 0.09]; AUC-ROC of the e-NIHSS for the second observer, 0.892 [95% CI 0.848–0.936, *p* = 0.01]) and PC strokes (AUC-ROC of the classical NIHSS, 0.804 [95% CI 0.689–0.920]; AUC-ROC of the e-NIHSS for the first observer, 0.800 [95% CI 0.685–0.916, *p* = 0.90]; AUC-ROC of the e-NIHSS for the second observer, 0.791 [95% CI 0.672–0.910, *p* = 0.68]) (Supplementary Figure 1).

**Figure 2:**
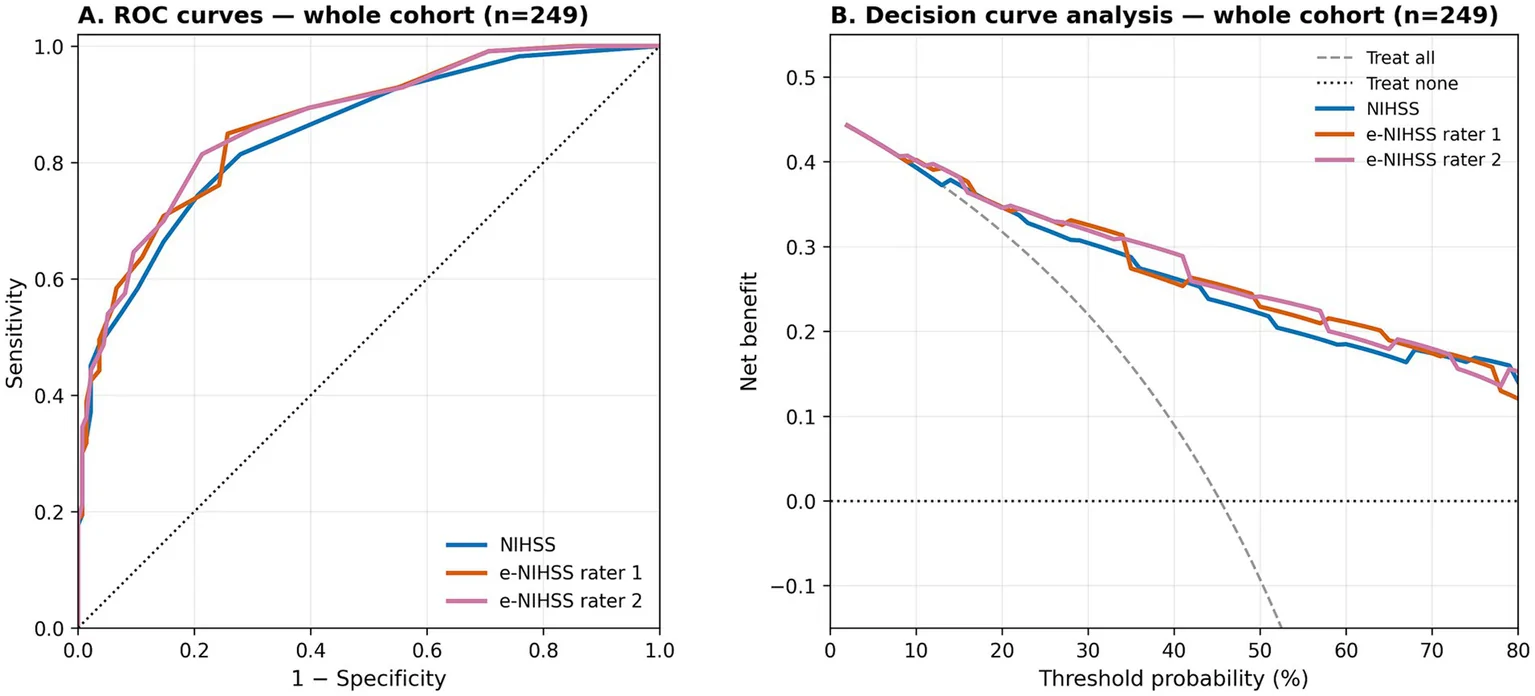
Discrimination and clinical net benefit of the Expanded National Institutes of Health Stroke Scale (e-NIHSS) versus the classical National Institutes of Health Stroke Scale (NIHSS) for predicting 3-month unfavorable outcome (modified Rankin Scale 3–6) in the whole cohort (*n* = 249). **(A)** Receiver operating characteristic (ROC) curves for the NIHSS and the e-NIHSS scored independently by two raters. **(B)** Decision curve analysis showing net benefit across threshold probabilities of 2–80%, referenced against the “treat all” and “treat none” strategies. Predicted probabilities for both panels were obtained from univariable logistic regression models using the NIHSS or the e-NIHSS as the sole predictor.

In the whole cohort, the e-NIHSS showed improved reclassification and discrimination relative to the NIHSS for both observers, reaching statistical significance for observer 2 (NRI + 0.881, 95% CI 0.246–1.203; IDI + 0.043, 95% CI 0.015–0.073) and a positive but more marginal effect for observer 1 (NRI + 0.727, 95% CI − 0.111-1.108; IDI + 0.032, 95% CI 0.0004–0.065) (Table 4). This pattern was replicated and reached significance for observer 2 in the AC stroke subgroup (Table 4). Calibration was adequate (Hosmer–Lemeshow *p* > 0.07) for both scores in nearly all strata, with the exception of the e-NIHSS for observer 1 in the whole cohort (χ^2^ = 25.73, df = 8, *p* = 0.001), attributable to a single small risk stratum (n = 12) with closely clustered predicted probabilities rather than a broad miscalibration pattern (Supplementary Table 4). DCA showed net benefit equal to or greater for the e-NIHSS than for the NIHSS across the clinically plausible 20–40% threshold range in the whole cohort (Figure 2B, Supplementary Table 5) and in AC strokes (Supplementary Table 5, Supplementary Figure 2) for both observers. In the PC stroke subgroup, however, none of the analyses demonstrated significantly better discriminative performance of the e-NIHSS than the classical NIHSS in predicting unfavorable functional outcome.

**Table 4:** Continuous net reclassification improvement and integrated discrimination improvement for e-NIHSS versus NIHSS predicting 3-month unfavorable outcome by observer and subgroup.

Rater	Subgroup (n)	AUC-ROC NIHSS → e-NIHSS	Continuous NRI (95% CI)	IDI (95% CI)
Observer 1	Whole cohort (*n* = 249)	0.849 → 0.867	+0.727 (−0.111 to +1.108)	+0.032 (+0.0004 to +0.065)
Observer 1	AC stroke (*n* = 194)	0.870 → 0.886	+0.623 (−0.167 to +1.284)	+0.029 (−0.007 to +0.066)
Observer 1	PC stroke (*n* = 55)	0.804 → 0.800	−0.089 (−0.988 to +0.907)	+0.001 (−0.097 to +0.092)
Observer 2	Whole cohort (*n* = 249)	0.849 → 0.871	+0.881 (+0.246 to +1.203)	+0.043 (+0.015 to +0.073)
Observer 2	AC stroke (*n* = 194)	0.870 → 0.892	+0.945 (+0.131 to +1.402)	+0.045 (+0.013 to +0.079)
Observer 2	PC stroke (*n* = 55)	0.804 → 0.791	−0.089 (−1.022 to +0.854)	−0.007 (−0.120 to +0.092)

AC, Anterior Circulation; AUC-ROC, area under the receiver operating characteristic curve; CI, Confidence Interval; e-NIHSS, Expanded National Institutes of Health Stroke Scale; ICC, intraclass correlation coefficient; IDI, integrated discrimination improvement; NIHSS, National Institutes of Health Stroke Scale; NRI, net reclassification improvement; PC, Posterior Circulation.

## 4 Discussion

We evaluated the diagnostic performance of the e-NIHSS for clinical assessment and outcome prediction in a cohort of patients with AC and PC strokes. The e-NIHSS was elaborated to overcome one of the main limitations of the NIHSS, that is, the underestimation of clinical severity in PC strokes, while retaining all its strengths, in particular, the ability to predict 3-month functional outcome. The e-NIHSS showed good predictive value, comparable to the classical NIHSS, for both AC and PC strokes. Moreover, the e-NIHSS was confirmed in a larger cohort of patients with acute stroke to be a quick scale, with excellent inter-rater reliability between trained examiners. The main advantage of the e-NIHSS over the classical NIHSS is its capacity to capture and quantify additional clinical signs relevant to PC strokes that are not fully represented in the classical NIHSS. The e-NIHSS score was higher than the classical NIHSS score in 75% of patients, with an average score increased by about 1.40 points. This difference was more pronounced in patients with PC strokes due to the higher score in the expanded items. In particular, nystagmus and/or Horner’s syndrome (expanded item 4) were more often present in PC strokes than in AC strokes. Thanks to these expanded items, the e-NIHSS captures and quantifies additional PC-relevant clinical signs that are not fully represented in the classical NIHSS. On the other hand, trunk ataxia (expanded item 11) was a very frequent finding in both AC and PC strokes, and it could be a useful item to evaluate stroke severity independently of stroke territory. The routine use of the e-NIHSS could therefore allow more complete capture of clinical signs in potentially disabling strokes. Whether this improved detection translates into a measurable increase in access to reperfusion treatments (i.e., IVT and/or EVT), however, was not directly evaluated in the present study and should be tested prospectively, for example by comparing treatment eligibility decisions made under each scoring system.

PC strokes represent about 20% of all strokes, but they are underrepresented in clinical studies for many reasons, including worse outcome and uncertain therapeutic benefits, as well as the complexity of their clinical presentation and the absence of available clinical scales for early identification and severity assessment of this stroke subtype. Therefore, a standardized, more sensitive evaluation of PC strokes with the e-NIHSS could facilitate the early identification of PC strokes and their inclusion in clinical studies.

Specific scales for PC strokes have been proposed, such as the Israeli Vertebrobasilar Stroke Scale (IVBSS) (16), the DEFENSIVE Stroke Scale (17), and, more recently, the Posterior NIHSS (POST-NIHSS) (18). The IVBSS is a 44-point and 11-item score elaborated in a small cohort of patients with PC stroke. The DEFENSIVE Stroke Scale is a 3-item scale developed for the identification of PC strokes by Emergency Department Physicians among patients presenting with acute-onset dizziness. Neither the IVBSS nor the DEFENSIVE Stroke Scale represents alternatives to the NIHSS, but are proposed as additional scales for the identification of patients with PC stroke, with the risk of prolonging clinical evaluation and delaying therapeutic decisions. The POST-NIHSS is a modified version of the NIHSS calculated by adding 3 items (gait/truncal ataxia, 3 points; dysphagia, 4 points; and abnormal cough, 5 points) to the baseline scale. This scale was demonstrated to improve prognostic accuracy in patients with PC stroke presenting with mild–moderate symptoms. However, only patients with PC stroke, NIHSS <10, and not treated with reperfusion therapies (i.e., IVT and/or EVT) were included. Moreover, inter-rater reliability was not evaluated. The applicability of these scales only to PC strokes limits their usefulness in the first evaluation of patients presenting with acute stroke when the involvement of the anterior or posterior circulation is still not known. Both the IVBSS and the POST-NIHSS include dysphagia testing, which could add more complexity to the neurological assessment, and be time-consuming and not so feasible in patients presenting to the Emergency Department with acute stroke. Finally, all these scales have a different structure in terms of the number of items and maximum score in comparison to the NIHSS. Conversely, the e-NIHSS keeps the 15-item and 42-point structure of the NIHSS unchanged, and allows, through the execution of a few additional commands, to better identify the clinical severity of patients with PC strokes. Whether this translates into a measurable impact on therapeutic strategies, such as eligibility for or use of reperfusion treatments, remains a clinically plausible hypothesis that requires prospective validation. The e-NIHSS proved to be a reliable tool, easy to administer to acute stroke patients, and with good discriminative performance to predict functional outcome, and could be a valid alternative to the classical NIHSS for the clinical assessment of both AC and PC stroke. This may be a particular strength in the Emergency Room, where a severity scale applicable to all strokes is required.

Our study has several limitations. First, it is based on observational data prospectively collected in a single Stroke Center, and external validation in a completely different cohort may be required. Second, the e-NIHSS was elaborated with an arbitrary experience-based scoring system characterized by an *a priori* choice of items and scores. This choice was made to expand some items of the NIHSS, but, at the same time, maintain the same number of items and the same range of scores of the classical scale. Third, clinical assessment with NIHSS and e-NIHSS was performed within 48 h of symptom onset, and we have no data on the distribution of assessment time within this relatively wide time window. This is a particularly relevant limitation for posterior-circulation stroke, where dynamic manifestations such as ataxia, cranial nerve deficits, and fluctuating consciousness may evolve substantially within this window; assessment timing could therefore have contributed to measurement variability in this subgroup, and future studies should record and analyze the exact time from symptom onset to assessment. Fourth, the discriminative performance to predict the 3-month functional outcome of the e-NIHSS for PC strokes was comparable to but not higher than the classical NIHSS. A possible explanation may be the relatively small number of patients with PC stroke in our study. In this regard, further validation in larger cohorts of PC stroke may be warranted. Consistent with this limitation, additional analyses performed to further quantify the incremental value of the e-NIHSS over the classical NIHSS converged on the same conclusion: a modest but reproducible improvement over the NIHSS in reclassification, discrimination, and clinical net benefit was demonstrated in the whole cohort and in AC strokes, but not in PC strokes, where confidence intervals were wide and compatible with a range of true effects, from a moderate benefit to a moderate harm, rather than with definitive evidence of no effect. We therefore consider the PC stroke subgroup analysis underpowered rather than genuinely null, and we revise our interpretation of the e-NIHSS’s value in PC stroke accordingly: the present data support its role in capturing and quantifying neurological deficits that the standard NIHSS is known to underweight, but do not yet establish an improvement in prognostic prediction specific to this subgroup. This distinction between detection validity and prognostic validity should be tested prospectively in a larger, adequately powered PC stroke cohort. A further methodological caveat applies to the calibration analysis specifically: because calibration intercept and slope were computed on the same sample used to derive each model, they are close to their trivial values (0 and 1, respectively) by construction, and the Hosmer–Lemeshow test — rather than intercept/slope — provided the main evidence used above. A fully rigorous calibration assessment would require external validation in an independent cohort or a bootstrap-corrected internal validation procedure, which was beyond the scope of the present single-cohort dataset and should be a priority for future validation of the e-NIHSS. We also emphasize that a non-significant Hosmer–Lemeshow test does not, by itself, establish adequate calibration, particularly in the relatively small PC subgroup (*n* = 55), where the test has limited power to detect miscalibration; the calibration, discrimination, and net-benefit findings reported throughout this manuscript should therefore be interpreted as exploratory, apparent-performance estimates rather than as evidence of validated model performance.

## 5 Conclusion

Compared to the classical NIHSS, the e-NIHSS showed similar discriminative performance in predicting 3-month functional outcome, with a modest incremental prognostic value in the whole cohort and in AC strokes, an effect more consistently supported by statistical significance for the second observer than for the first. In PC strokes specifically, the e-NIHSS was not shown to improve prognostic prediction beyond the classical NIHSS at the sample size available, but demonstrated its capacity to capture and quantify additional signs and symptoms of PC stroke, along with excellent inter-rater reliability. Given these characteristics, the e-NIHSS demonstrated good diagnostic performance and may be a valid tool for the clinical assessment of all patients presenting with symptoms of acute stroke, while its incremental prognostic value in PC stroke specifically warrants confirmation in larger, adequately powered cohorts.

## Data Availability

The raw data supporting the conclusions of this article will be made available by the authors, without undue reservation.

## In memoriam

MC was the creator of the e-NIHSS, and she has led research on this topic over the years, including this manuscript. We hope to be up to par to continue the work. This manuscript is dedicated to MC for everything she taught us as persons and as doctors. Her humanity, moral integrity, and passion for research and patients will always guide us.
