# Supplementary material for: Diagnostic performance of the expanded National Institutes of Health Stroke Scale for the assessment of anterior and posterior circulation strokes
Source: Front Neurol. 2026 Sep 14;17:1788839. doi: 10.3389/fneur.2026.1788839 (PMC13616663; doi:10.3389/fneur.2026.1788839)
Supplement: Supplementary file 1 [file Supplementary_file_1.pdf]

Supplementary Table 1 (A). Univariate and multivariate logistic regression analyses for unfavorable functional outcome (i.e., mRS 3-6) at 3 months for all strokes using the classical NIHSS.

	**Univariate analysis**		**Multivariate analysis**	
	**OR (95% CI)**	**p value**	**OR (95% CI)**	**p value**
**NIHSS (per 1 point)**	**1.385 (1.263-1.518)**	**<0.001**	**1.464 (1.306-1.642)**	**<0.001**
Age (per year)	1.063 (1.038-1.088)	<0.001	1.068 (1.029-1.109)	0.001
Male sex	0.491 (0.296-0.816)	0.006	0.378 (0.177-0.809)	0.012
Atrial fibrillation	2.574 (1.461-4.535)	0.001	1.360 (0.601-3.078)	0.461
Hypertension	1.942 (1.006-3.752)	0.048	1.038 (0.392-2.747)	0.941
Diabetes mellitus	2.126 (1.118-4.043)	0.021	2.883 (1.163-7.145)	0.022
Hypercholesterolemia	1.128 (0.680-1.870)	0.642	/	/
Current smoking	0.439 (0.235-0.818)	0.010	0.635 (0.243-1.663)	0.356
Coronary artery disease	1.399 (0.729-2.688)	0.313	/	/
Previous stroke/TIA	0.832 (0.306-2.261)	0.719	/	/
Hemorrhagic stroke	2.297 (1.043-5.058)	0.039	0.906 (0.270-3.036)	0.873
Intravenous thrombolysis	0.455 (0.272-0.760)	0.003	0.449 (0.206-0.977)	0.043
Mechanical thrombectomy	1.524 (0.889-2.610)	0.125	/	/

Supplementary Table 1 (B). Univariate and multivariate logistic regression analyses for unfavorable functional outcome (i.e., mRS 3-6) at 3 months for all strokes using the e-NIHSS for the first observer.

	**Univariate analysis**		**Multivariate analysis**	
	**OR (95% CI)**	**p value**	**OR (95% CI)**	**p value**
**e-NIHSS observer 1 (per 1 point)**	**1.370 (1.257-1.494)**	**<0.001**	**1.435 (1.291-1.596)**	**<0.001**
Age (per year)	1.063 (1.038-1.088)	<0.001	1.070 (1.030-1.112)	<0.001
Male sex	0.491 (0.296-0.816)	0.006	0.367 (0.169-0.798)	0.011
Atrial fibrillation	2.574 (1.461-4.535)	0.001	1.320 (0.575-3.032)	0.512
Hypertension	1.942 (1.006-3.752)	0.048	0.955 (0.352-2.592)	0.927
Diabetes mellitus	2.126 (1.118-4.043)	0.021	2.350 (0.943-5.859)	0.067
Hypercholesterolemia	1.128 (0.680-1.870)	0.642	/	/
Current smoking	0.439 (0.235-0.818)	0.010	0.627 (0.233-1.688)	0.355
Coronary artery disease	1.399 (0.729-2.688)	0.313	/	/
Previous stroke/TIA	0.832 (0.306-2.261)	0.719	/	/
Hemorrhagic stroke	2.297 (1.043-5.058)	0.039	0.792 (0.226-2.778)	0.715
Intravenous thrombolysis	0.455 (0.272-0.760)	0.003	0.493 (0.223-1.091)	0.081
Mechanical thrombectomy	1.524 (0.889-2.610)	0.125	/	/

Supplementary Table 1 (C). Univariate and multivariate logistic regression analyses for unfavorable functional outcome (i.e., mRS 3-6) at 3 months for all strokes using the e-NIHSS for the second observer.

	**Univariate analysis**		**Multivariate analysis**	
	**OR (95% CI)**	**p value**	**OR (95% CI)**	**p value**
**e-NIHSS observer 2 (per 1 point)**	**1.395 (1.273-1.527)**	**<0.001**	**1.460 (1.306-1.632)**	**<0.001**
Age (per year)	1.063 (1.038-1.088)	<0.001	1.067 (1.027-1.108)	0.001
Male sex	0.491 (0.296-0.816)	0.006	0.357 (0.162-0.784)	0.010
Atrial fibrillation	2.574 (1.461-4.535)	0.001	1.321 (0.567-3.079)	0.519
Hypertension	1.942 (1.006-3.752)	0.048	1.090 (0.395-3.009)	0.868

Diabetes mellitus	2.126 (1.118-4.043)	0.021	2.301 (0.912-5.801)	0.077
Hypercholesterolemia	1.128 (0.680-1.870)	0.642	/	/
Current smoking	0.439 (0.235-0.818)	0.010	0.625 (0.231-1.686)	0.353
Coronary artery disease	1.399 (0.729-2.688)	0.313	/	/
Previous stroke/TIA	0.832 (0.306-2.261)	0.719	/	/
Hemorrhagic stroke	2.297 (1.043-5.058)	0.039	0.837 (0.234-2.995)	0.784
Intravenous thrombolysis	0.455 (0.272-0.760)	0.003	0.484 (0.217-1.081)	0.077
Mechanical thrombectomy	1.524 (0.889-2.610)	0.125	/	/

Supplementary Table 2 (A). Univariate and multivariate logistic regression analyses for unfavorable functional outcome (i.e., mRS 3-6) at 3 months for anterior circulation strokes using the classical NIHSS.

	**Univariate analysis**		**Multivariate analysis**	
	**OR (95% CI)**	**p value**	**OR (95% CI)**	**p value**
**NIHSS (per 1 point)**	**1.394 (1.260-1.542)**	**<0.001**	**1.450 (1.284-1.637)**	**<0.001**
Age (per year)	1.060 (1.033-1.089)	<0.001	1.068 (1.021-1.116)	0.004
Male sex	0.502 (0.283-0.892)	0.019	0.408 (0.173-0.959)	0.040
Atrial fibrillation	2.204 (1.174-4.138)	0.014	1.575 (0.634-3.910)	0.328
Hypertension	1.587 (0.765-3.294)	0.215	/	/
Diabetes mellitus	2.754 (1.213-6.252)	0.015	2.965 (0.943-9.320)	0.063
Hypercholesterolemia	1.046 (0.584-1.874)	0.879	/	/
Current smoking	0.478 (0.242-0.944)	0.033	1.019 (0.355-2.926)	0.972
Coronary artery disease	1.135 (0.536-2.402)	0.741	/	/
Previous stroke/TIA	1.012 (0.327-3.130)	0.983	/	/
Hemorrhagic stroke	1.732 (0.751-3.995)	0.197	/	/
Intravenous thrombolysis	0.445 (0.248-0.797)	0.006	0.588 (0.254-1.361)	0.215
Mechanical thrombectomy	1.414 (0.782-2.557)	0.251	/	/

Supplementary Table 2 (B). Univariate and multivariate logistic regression analyses for unfavorable functional outcome (i.e., mRS 3-6) at 3 months for anterior circulation strokes using the e-NIHSS for the first observer.

	**Univariate analysis**		**Multivariate analysis**	
	**OR (95% CI)**	**p value**	**OR (95% CI)**	**p value**
**e-NIHSS observer 1 (per 1 point)**	**1.371 (1.248-1.507)**	**<0.001**	**1.412 (1.266-1.574)**	**<0.001**
Age (per year)	1.060 (1.033-1.089)	<0.001	1.069 (1.022-1.119)	0.004
Male sex	0.502 (0.283-0.892)	0.019	0.414 (0.174-0.986)	0.046
Atrial fibrillation	2.204 (1.174-4.138)	0.014	1.570 (0.621-3.972)	0.341
Hypertension	1.587 (0.765-3.294)	0.215	/	/
Diabetes mellitus	2.754 (1.213-6.252)	0.015	2.802 (0.859-9.141)	0.088
Hypercholesterolemia	1.046 (0.584-1.874)	0.879	/	/
Current smoking	0.478 (0.242-0.944)	0.033	1.039 (0.350-3.082)	0.946
Coronary artery disease	1.135 (0.536-2.402)	0.741	/	/
Previous stroke/TIA	1.012 (0.327-3.130)	0.983	/	/
Hemorrhagic stroke	1.732 (0.751-3.995)	0.197	/	/
Intravenous thrombolysis	0.445 (0.248-0.797)	0.006	0.623 (0.264-1.473)	0.281

Mechanical thrombectomy	1.414 (0.782-2.557)	0.251	/	/

Supplementary Table 2 (C). Univariate and multivariate logistic regression analyses for unfavorable functional outcome (i.e., mRS 3-6) at 3 months for anterior circulation strokes using the e-NIHSS for the second observer.

	**Univariate analysis**		**Multivariate analysis**	
	**OR (95% CI)**	**p value**	**OR (95% CI)**	**p value**
**e-NIHSS observer 2 (per 1 point)**	**1.396 (1.265-1.540)**	**<0.001**	**1.445 (1.285-1.624)**	**<0.001**
Age (per year)	1.060 (1.033-1.089)	<0.001	1.066 (1.018-1.117)	0.006
Male sex	0.502 (0.283-0.892)	0.019	0.385 (0.157-0.939)	0.036
Atrial fibrillation	2.204 (1.174-4.138)	0.014	1.621 (0.629-4.176)	0.317
Hypertension	1.587 (0.765-3.294)	0.215	/	/
Diabetes mellitus	2.754 (1.213-6.252)	0.015	2.845 (0.848-9.543)	0.090
Hypercholesterolemia	1.046 (0.584-1.874)	0.879	/	/
Current smoking	0.478 (0.242-0.944)	0.033	0.980 (0.325-2.953)	0.971
Coronary artery disease	1.135 (0.536-2.402)	0.741	/	/
Previous stroke/TIA	1.012 (0.327-3.130)	0.983	/	/
Hemorrhagic stroke	1.732 (0.751-3.995)	0.197	/	/
Intravenous thrombolysis	0.445 (0.248-0.797)	0.006	0.612 (0.254-1.475)	0.274
Mechanical thrombectomy	1.414 (0.782-2.557)	0.251	/	/

Supplementary Table 3 (A). Univariate and multivariate logistic regression analyses for unfavorable functional outcome (i.e., mRS 3-6) at 3 months for posterior circulation strokes using the classical NIHSS.

	**Univariate analysis**		**Multivariate analysis**	
	**OR (95% CI)**	**p value**	**OR (95% CI)**	**p value**
**NIHSS (per 1 point)**	**1.548 (1.178-2.034)**	**0.002**	**1.664 (1.218-2.272)**	**0.001**
Age (per year)	1.072 (1.018-1.128)	0.009	1.098 (1.022-1.180)	0.011
Male sex	0.451 (0.152-1.337)	0.151	/	/
Atrial fibrillation	4.821 (1.279-18.178)	0.020	1.413 (0.260-7.688)	0.689
Hypertension	4.500 (0.871-23.250)	0.073	/	/
Diabetes mellitus	1.467 (0.472-4.555)	0.508	/	/
Hypercholesterolemia	1.562 (0.528-4.628)	0.421	/	/
Current smoking	0.261 (0.050-1.369)	0.112	/	/
Coronary artery disease	2.779 (0.706-10.940)	0.144	/	/
Previous stroke/TIA	0.406 (0.040-4.168)	0.448	/	/
Hemorrhagic stroke	-	0.999	/	/
Intravenous thrombolysis	0.494 (0.166-1.466)	0.204	/	/
Mechanical thrombectomy	2.250 (0.556-9.112)	0.256	/	/

Supplementary Table 3 (B). Univariate and multivariate logistic regression analyses for unfavorable functional outcome (i.e., mRS 3-6) at 3 months for posterior circulation strokes using the e-NIHSS for the first observer.

	**Univariate analysis**		**Multivariate analysis**	
	**OR (95% CI)**	**p value**	**OR (95% CI)**	**p value**

**e-NIHSS observer 1 (per 1 point)**	**1.442 (1.150-1.810)**	**0.002**	**1.589 (1.203-2.099)**	**0.001**
Age (per year)	1.072 (1.018-1.128)	0.009	1.107 (1.027-1.194)	0.008
Male sex	0.451 (0.152-1.337)	0.151	/	/
Atrial fibrillation	4.821 (1.279-18.178)	0.020	1.194 (0.217-6.564)	0.839
Hypertension	4.500 (0.871-23.250)	0.073	/	/
Diabetes mellitus	1.467 (0.472-4.555)	0.508	/	/
Hypercholesterolemia	1.562 (0.528-4.628)	0.421	/	/
Current smoking	0.261 (0.050-1.369)	0.112	/	/
Coronary artery disease	2.779 (0.706-10.940)	0.144	/	/
Previous stroke/TIA	0.406 (0.040-4.168)	0.448	/	/
Hemorrhagic stroke	-	0.999	/	/
Intravenous thrombolysis	0.494 (0.166-1.466)	0.204	/	/
Mechanical thrombectomy	2.250 (0.556-9.112)	0.256	/	/

Supplementary Table 3 (C). Univariate and multivariate logistic regression analyses for unfavorable functional outcome (i.e., mRS 3-6) at 3 months for posterior circulation strokes using the e-NIHSS for the second observer.

	**Univariate analysis**		**Multivariate analysis**	
	**OR (95% CI)**	**p value**	**OR (95% CI)**	**p value**
**e-NIHSS observer 2 (per 1 point)**	**1.471 (1.154-1.874)**	**0.002**	**1.600 (1.186-2.158)**	**0.002**
Age (per year)	1.072 (1.018-1.128)	0.009	1.098 (1.023-1.178)	0.009
Male sex	0.451 (0.152-1.337)	0.151	/	/
Atrial fibrillation	4.821 (1.279-18.178)	0.020	1.185 (0.215-6.524)	0.845
Hypertension	4.500 (0.871-23.250)	0.073	/	/
Diabetes mellitus	1.467 (0.472-4.555)	0.508	/	/
Hypercholesterolemia	1.562 (0.528-4.628)	0.421	/	/
Current smoking	0.261 (0.050-1.369)	0.112	/	/
Coronary artery disease	2.779 (0.706-10.940)	0.144	/	/
Previous stroke/TIA	0.406 (0.040-4.168)	0.448	/	/
Hemorrhagic stroke	-	0.999	/	/
Intravenous thrombolysis	0.494 (0.166-1.466)	0.204	/	/
Mechanical thrombectomy	2.250 (0.556-9.112)	0.256	/	/

Supplementary Table 4. Calibration (Hosmer–Lemeshow goodness-of-fit test) for NIHSS and e-NIHSS models predicting 3-month unfavorable outcome (mRS 3–6), by rater and subgroup.

Rater	Subgroup (n)	Score	HL χ² (df)	HL p-value
Rater 1	Whole cohort (n=249)	NIHSS	7.14 (7)	0.415
Rater 1	Whole cohort (n=249)	e-NIHSS	25.73 (8)	0.001
Rater 1	Anterior circulation (n=194)	NIHSS	5.73 (7)	0.572
Rater 1	Anterior circulation (n=194)	e-NIHSS	11.85 (8)	0.158
Rater 1	Posterior circulation (n=55)	NIHSS	0.45 (3)	0.929
Rater 1	Posterior circulation (n=55)	e-NIHSS	0.51 (4)	0.973
Rater 2	Whole cohort (n=249)	NIHSS	7.14 (7)	0.415
Rater 2	Whole cohort (n=249)	e-NIHSS	14.41 (8)	0.072
Rater 2	Anterior circulation (n=194)	NIHSS	5.73 (7)	0.572
Rater 2	Anterior circulation (n=194)	e-NIHSS	9.58 (8)	0.296
Rater 2	Posterior circulation (n=55)	NIHSS	0.45 (3)	0.929
Rater 2	Posterior circulation (n=55)	e-NIHSS	0.68 (4)	0.954

Abbreviations: HL = Hosmer–Lemeshow; df = degrees of freedom. Calibration was assessed using univariable logistic regression models with either NIHSS or e-NIHSS as the sole predictor of 3-month unfavorable outcome.

Supplementary Table 5. Difference in net benefit (e-NIHSS minus NIHSS) at 20%, 30%, and 40% decision thresholds for predicting 3-month unfavorable outcome (mRS 3–6), by rater and subgroup.

Rater	Subgroup (n)	ΔNB @20%	ΔNB @30%	ΔNB @40%
Rater 1	Whole cohort (n=249)	+0.0000	+0.0212	−0.0054
Rater 1	Anterior circulation (n=194)	+0.0142	+0.0265	+0.0137
Rater 1	Posterior circulation (n=55)	−0.0045	+0.0000	+0.0061
Rater 2	Whole cohort (n=249)	−0.0010	+0.0149	+0.0295
Rater 2	Anterior circulation (n=194)	+0.0090	+0.0272	+0.0137
Rater 2	Posterior circulation (n=55)	−0.0045	+0.0182	−0.0303

Abbreviations: NB = net benefit; ΔNB = difference in net benefit (e-NIHSS minus NIHSS). Net benefit was calculated as (true positives/n) – (false positives/n) × [threshold/(1−threshold)], using the same univariable logistic regression models described for Supplementary Table 4. Positive values indicate e-NIHSS provided greater net benefit than NIHSS at that threshold.

Supplementary Figure 1. ROC curves for NIHSS versus e-NIHSS predicting 3-month unfavorable outcome (mRS 3–6), by rater and subgroup.

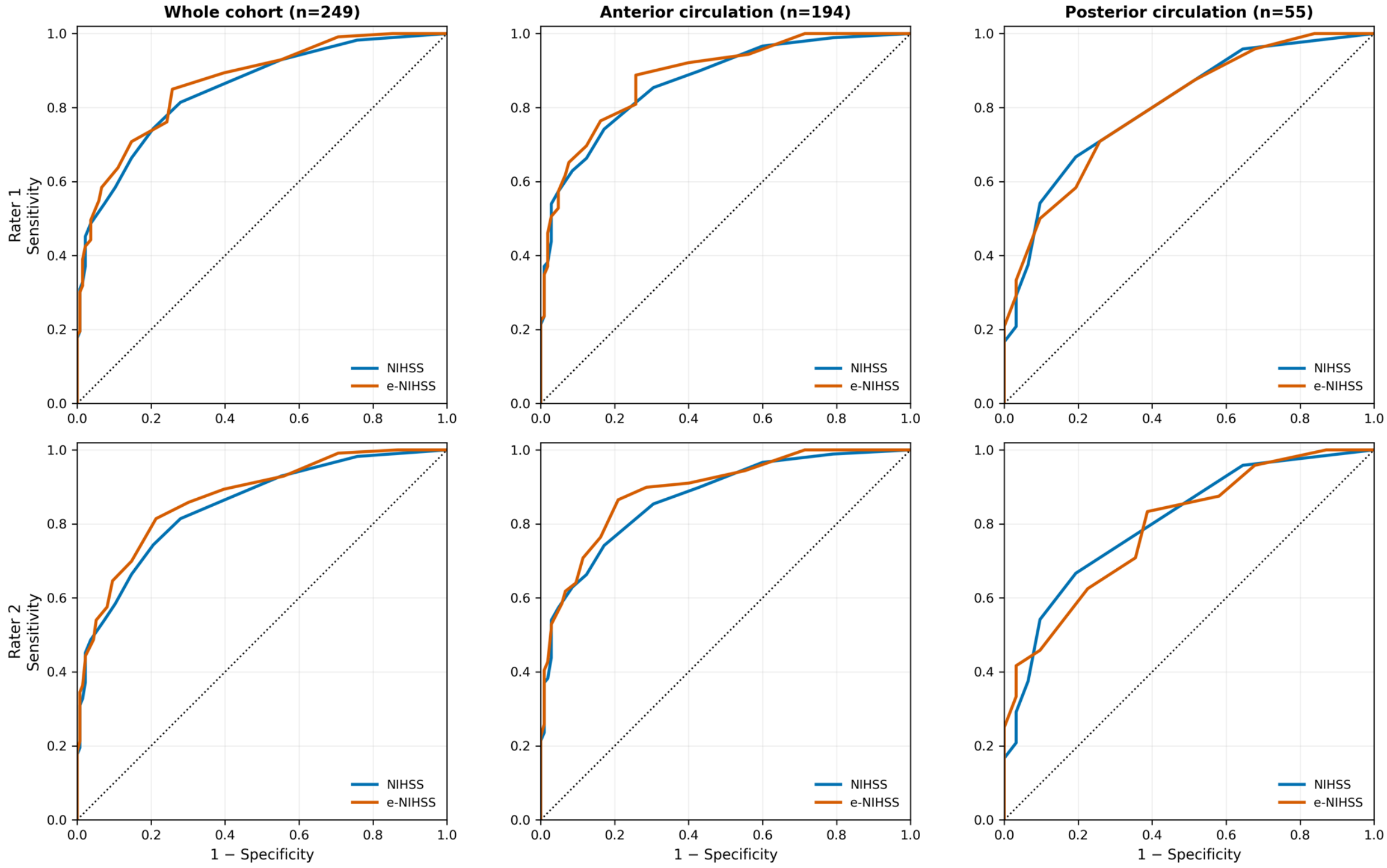

Rows: rater 1 (top) and rater 2 (bottom). Columns: whole cohort, anterior-circulation, and posterior-circulation subgroups. Diagonal dotted line represents chance discrimination (AUC = 0.5). Blue: NIHSS; orange: e-NIHSS.

Supplementary Figure 2. Decision curve analysis comparing NIHSS and e-NIHSS for predicting 3-month unfavorable outcome (mRS 3–6), by rater and subgroup.

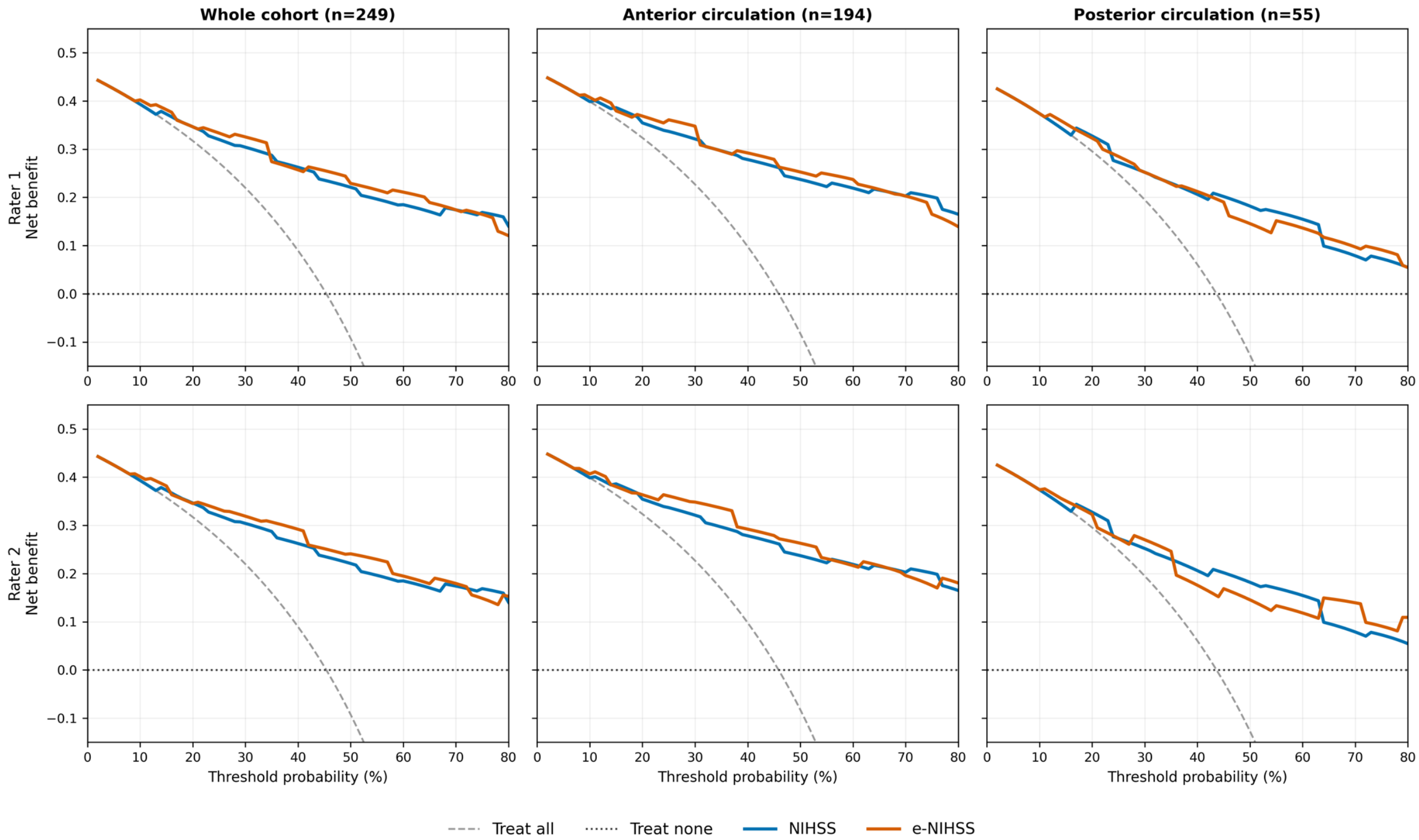

Rows: rater 1 (top) and rater 2 (bottom). Columns: whole cohort, anterior-circulation, and posterior-circulation subgroups. Grey dashed line: "treat all" strategy; black dotted line: "treat none" strategy (net benefit = 0). Blue: NIHSS; orange: e-NIHSS.
